# Management of intrahepatic recurrence after resection for hepatocellular carcinoma exceeding the barcelona clinic liver cancer criteria

**DOI:** 10.18632/oncotarget.22779

**Published:** 2017-11-30

**Authors:** Wei Xu, Rui Guo, Gang Xu, Lejia Sun, Dandan Hu, Haifeng Xu, Huayu Yang, Xinting Sang, Xin Lu, Yilei Mao

**Affiliations:** ^1^ Professor of Surgery, Department of Liver Surgery, Peking Union Medical College Hospital, Chinese Academy of Medical Sciences and Peking Union Medical College, Beijing, China; ^2^ Professor of Surgery, Department of Surgery, Peking Plastic Surgery Hospital, Chinese Academy of Medical Sciences and Peking Union Medical College, Beijing, China

**Keywords:** hepatocellular carcinoma, hepatectomy, clinical stage, early recurrence, prognosis

## Abstract

**Background:**

Although patients with Barcelona clinic liver cancer stage B or C hepatocellular carcinoma derive survival benefit from hepatectomy, prognostic factors and management after curative resection are unclear. This study aims to evaluate predictive factors, therapy and prognosis of intra-hepatic recurrences after curative resection of Barcelona clinic liver cancer stage B or C hepatocellular carcinoma.

**Methods:**

We retrospectively analyzed 397 patients with Barcelona clinic liver cancer stage B or C hepatocellular carcinoma who underwent curative resections from January 1989 to October 2011. Intra-hepatic recurrences were classified into early (<2 year) and late (≥2 year) recurrences.

**Results:**

Overall survival rates in our cohort were 1-year: 81.4%; 3-year: 48.5%; and 5-year: 28.2%. Early and late intra-hepatic recurrences developed in 104 patients and 73 patients, respectively. In univariate analysis, overall survival for the non-recurrence group was significantly better than for the recurrence group (*P*<0.001), and overall survival for the late recurrence group was significantly better than for the early recurrence group (*P*<0.001). In multivariate analysis, total tumor size, tumor number and vascular invasion were significant risk factors for tumor recurrence (*P*<0.001). The overall survival of patients with late recurrence who received curative treatment was comparable to those who never had tumor recurrences (*P*=0.140).

**Conclusion:**

Time to recurrence and feasibility of curative treatment are the best determinants for prognosis in Barcelona clinic liver cancer stage B or C hepatocellular carcinoma. Curative treatments may prolong overall survival of patients with late recurrences, but should be avoided for those with early recurrences.

## INTRODUCTION

Hepatocellular carcinoma (HCC) ranks fifth in cancer incidence and third in cancer mortality worldwide [1]. The Barcelona Clinic Liver Cancer (BCLC) guideline is a commonly used staging system that accounts for tumor burden, liver function, and general HCC conditions, and assigns therapies accordingly [2]. According to the BCLC staging system, curative treatments, including hepatic resection and radiofrequency ablation (RFA), are only indicated for early-stage HCC, whereas patients with intermediate-stage disease (BCLC-B) are candidates only for palliative treatments (chemoembolization); and those with advanced HCC (BCLC-C) would only be treated by sorafenib [3]. However, in many trials, overall survival (OS) of patients with intermediate or advanced HCC after hepatic resection was longer than after palliative treatment, especially in Asia-Pacific studies [4–13].

Although surgical treatment improved the survival rate of patients with BCLC-B or -C stage HCC, its prognosis is still poor, especially in patients with recurrent tumors. We suppose that some of these patients could still undergo curative treatment, and selection of optimal post-surgical treatment might further improve their OS. Information that could improve predictions of their likely outcomes could facilitate appropriate management of these patients after their surgeries.

However, detailed analyses of large series that evaluate predictive factors, therapies, and prognosis of intrahepatic recurrence after surgery are not available because so few cases are found in clinical practice. We are unaware of any prognostic scores based on statistical analysis of patients with intermediate-stage HCC who received hepatectomy. Therefore, in the present study, we retrospectively analyzed the data of 397 consecutive patients with BCLC stage B/C HCC who were treated by hepatectomy in our institute, to investigate the prognostic factors influencing its recurrence and OS, and to evaluate the management of these patients after intra-hepatic recurrence.

## RESULTS

### Patients

Among the 1050 HCC patients who received partial hepatectomies with curative intent at PUMCH, 317 (30.2%) were in BCLC Stage B and 80 (7.6%) cases in BCLC Stage C. Among the 397 patients with BCLC B-C HCC, 282 had HBV infection and 39 had HCV infection. All patients had Child-Pugh grade A preoperative liver function and 73.6% HCC patients had liver cirrhosis of different degrees. None of the 80 patients with BCLC-C HCC had extrahepatic metastasis, but 14 had multiple tumors (Table 1).

**Table 1:** Baseline characteristics

characteristics	Barcelona clinic liver cancer stage B (n=317)	Barcelona clinic liver cancer stage C (n=80)	Total
Age (years)	56.54 ± 12.31	53.61 ± 11.27	55.95 ± 12.15
Gender (male/female)	263/54	71/9	334/63
ALT (IU/L) (A/N)	135/182	40/40	175/222
AST (IU/L) (A/N)	162/155	48/32	210/187
Total bilirubin(mmol/L) (A/N)	56/261	13/67	69/328
Albumin (g/L) (A/N)	45/272	16/63	61/335
Prothrombin time (s) (A/N)	105/212	26/54	131/266
Hemoglobin (g/L) (A/N)	39/278	14/66	53/344
Liver cirrhosis(Y/N)	223/94	69/11	292/105
AFP (A/N)	174/143	56/24	230/167
HBsAg (+)(Y/N)	227/90	55/25	282/115
HCV antibody(+) (Y/N)	30/287	9/71	39/358
Tumor size	6.61 ± 3.36	7.89 ± 4.24	6.87 ± 3.59
Multiple tumors (Y/N)	—	14/66	—
Tumor differentiation (III-IV/I-II)	81/236	30/50	111/286
Extent of portal vein invasion (I/II/III)	—	8/12/60	—
Extra-hepatic Spread (Y/N)	—	0/80	—

A/N: abnormal/normal; Y/N: Yes/No.

### Follow-up and prognostic factors for survival of HCC patients in BCLC stage B or C

**Figure 1:**
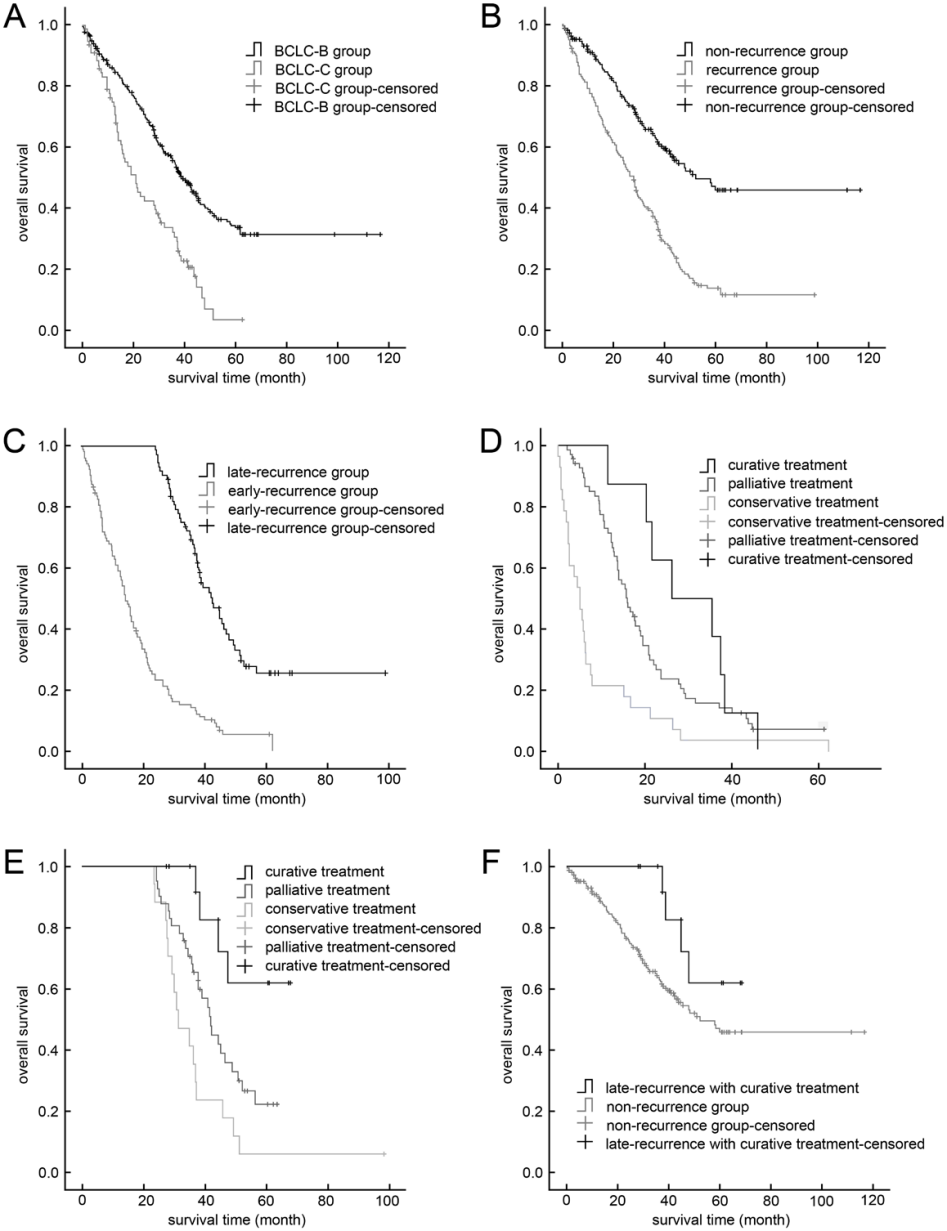
Kaplan–Meier curves **(A)** Stage BCLC-B hepatocellular carcinoma group vs stage BCLC-C group. **(B)** Recurrence vs. non-recurrence groups (*P*<0.001). **(C)** Late-recurrence vs. early-recurrence groups (*P*<0.001); **(D)** Early-recurrence group with curative treatment vs. palliative treatment (*P*=0.222) and vs. conservative treatment (*P*<0.001). **(E)** Late-recurrence group with curative treatment vs. palliative treatment (*P*=0.016) and vs. conservative treatment (*P*<0.001). **(F)** Late-recurrence group with curative treatment vs. non-recurrence group (*P*=0.140).

All patients in the BCLC Stage B/C cohort were followed-up until October 2016 (median follow-up time: 28 months; range: 1-117 months). Their OS rates were 1-year: 81.4%; 3-year: 48.5%; and 5-year: 28.2% and the perioperative death rate was 2.0% (8/397). The OS rates for the BCLC-B subgroup were 1-year: 85.3%; 3-year: 54.8%; and 5-year: 33.8% (perioperative death rate: 2.2% [7/317]); OS rates for the BCLC-C subgroup were 1-year: 73.5%; 3-year: 29.2%; and 5-year: 3.6%, (perioperative death rate: 1.3% [1/80]; Figure 1A). The DFS rates for the BCLC-B/C cohort were 1-year: 76.1%; 3-year: 40.5%; and 5-year: 23.4%. The DFS rates for the BCLC-B subgroup were 1-year: 78.1%; 3-year: 44.4%; and 5-year: 27.2%; DFS rates for the BCLC-C subgroup were 1-year: 68.4%; 3-year: 23.8%; and 5-year: 0%.

In univariate analysis, tumor size, tumor number, vascular invasion, Edmondson grade, liver cirrhosis, AFP level, HBsAg, AST and prothrombin time were prognostic factors for both OS and DFS of BCLC-B/C patients (Table 2). In multivariate analysis, ≥ 3 tumors, tumor ≥ 10 cm and vascular invasion were independent risk factors for both OS and DFS.

**Table 2:** Univariate and Multivariate analysis of risk factors related to OS and DFS of BCLC Stage B or C HCC patients

	OS				DFS			
	Median survival (month)	P value	HR (95% CI)	P value	Median survival (month)	P value	HR (95% CI)	P value
Age (>60/≤60 years)	37.2/34.8	0.398			30.8/24.6	0.431		
Gender (male/female)	35.7/36.7	0.830			29.0/28.0	0.630		
ALT (A/N)	31.6/37.5	0.225			22.5/31.3	0.064		
AST (A/N)	28.7/42.3	**0.002**	—	0.383	22.5/35.0	**0.006**	—	0.608
Total bilirubin (A/N)	35.7/36.2	0.437			27.3/28.6	0.220		
Albumin (A/N)	24.6/37.2	**0.012**	—	0.322	22.1/29.0	0.105		
Prothrombin time (A/N)	29.7/37.5	**0.007**	—	0.061	24.2/29.0	**0.006**	—	0.056
Hemoglobin (A/N)	31.1/36.7	0.601			28.1/28.3	0.895		
Liver cirrhosis(Y/N)	32.1/43.1	**0.012**	—	0.553	24.0/36.2	**0.002**	—	0.145
AFP (A/N)	29.7/44.7	**<0.001**	—	0.098	21.9/36.0	**0.001**	—	0.121
HBsAg (+)(Y/N)	31.5/37.9	**0.036**	—	0.311	23.0/35.0	**0.019**	—	0.310
HCV antibody(+) (Y/N)	36.5/36.0	0.945			29.0/28.1	0.573		
Tumor number (>3/≤3)	9.8/36.4	**0.009**	4.649 (1.454-14.806)	**0.010**	9.7/28.5	**0.038**	3.361 (1.055-10.705)	**0.040**
Tumor size (>10cm/≤10cm)	18.4/38.2	**0.000**	1.654 (1.196-2.288)	**0.002**	13.0/31.3	**0.000**	1.693 (1.229-2.333)	**0.001**
Edmondson grade (III–IV/ I–II)	28.5/40.8	**0.001**	—	0.122	19.1/31.3	**0.007**	—	0.400
Vascular invasion	20.8/39.3	**<0.001**	1.963 (1.432-2.691)	**0.001**	16.3/31.2	**<0.001**	1.558 (1.142-2.125)	**0.005**

A/N: abnormal/normal; Y/N: Yes/No.

### Therapy and prognosis after recurrence

Of the 397 BCLC-B/C patients, 177 patients (44.6%) developed intra-hepatic recurrences, including 104 early recurrences and 73 late recurrences. Their clinical data and management are summarized in Table 3. In the recurrence subgroup, OS rates were 1-year: 75.5%; 3-year: 36.6%; and 5-year: 6.1%—significantly worse than for the non-recurrence subgroup (1-year: 89.6%; 3-year: 62.9%; 5-year: 13.8%; *P*<0.001; Figure 1B). Overall survival was significantly better in the late-recurrence subgroup (1-year: 100%; 3-year: 67.7%; 5-year: 25.5%) than in early-recurrence subgroup (1-year: 58.0%; 3-year: 13.1%; 5-year: 5.4%; *P*<0.001; Figure 1C). Median time of early recurrence was 13.7 months, and of late recurrence was 38.1 months.

**Table 3:** Clinical data and management of HCC patients with early and late recurrences

Characteristics	Early recurrence (n=104)	Late recurrence (n=73)	*P* value
Time of recurrence(median, months)	7.8	37.3	—
Age (median, years)	54.11 ± 11.70	59.10 ± 11.04	0.005
Gender (male/female)	91/13	60/13	0.390
BCLC Stage			0.366
B (n/%)	77 (74.0)	59 (80.8)	
C (n/%)	27 (26.0)	14 (19.2)	
Overall survival (median, months)	13.7	38.1	**<0.001**
Treatment			**0.021**
Curative Treatment (n/%)	8 (7.7)	15 (20.5)	
Palliative Treatment (n/%)	96 (92.3)	58 (79.5)	—

Among the 177 patients in the recurrent subgroup, 18 patients underwent re-resection after recurrence, 4 received RFA, 98 received TACE, 11 received sorafenib, and the other 46 patients received conservative management. In the early-recurrence subgroup (n=104), treatment was feasible for only 8 patients (7.7%), including surgery (n=5) and RFA (n=2); but was possible for significantly more patients in the late-recurrence subgroup (n=15, 20.5%; *P*=0.021), including surgery (n=13) and RFA (n=2).

The 1-, 3-, and 5-year The OS rates of patients who received curative treatment for recurrence were 1-year: 91.3%; 3-year: 72.6%; and 5-year: 37.4%-significantly better than for those treated by other methods (1-year: 72.5%; 3-year: 30.6%; 5-year: 10.3%; *P*<0.001). Furthermore, in the early-recurrence subgroup, OS rates for those who received curative treatment for recurrences (1-year: 87.5%; 3-year: 37.5%; 5-year: 0%) did not significantly differ from those of patients who received palliative treatment only (1-year: 71.3%; 3-year: 23.6%; 5-year: 7.2%, *P*=0.222), though they were better than those who received conservative management after recurrence (1-year: 21.4%; 3-year: 3.6% 5-year: 0%,*P*<0.001, Figure 1D). In the late-recurrence subgroup, those who received curative treatments had better OS rates (1-year: 100%; 3-year: 91.7%; 5-year: 61.9%) than did those with palliative treatment (1-year: 100%; 3-year: 68.0%; 5-year: 22.1%; *P*=0.016) or conservative treatment (1-year:100%; 3-year:35.3%; 5-year: 5.9%; *P*<0.001, Figure 1E). Interestingly, the OS of the late-recurrence patients who received curative treatment was comparable to those who never had tumor recurrence (*P*=0.140, Figure 1F).

## DISCUSSION

Resection has been shown to have survival benefits for patients with BCLC-B/C HCC. For patients with BCLC-B HCC, liver resection is proven to be as safe as TACE and to provide better long-term overall outcomes [4–6], [9–11]; and for some BCLC-C HCC patients, surgical resection can offer lower mortality, acceptable morbidity and favorable survival benefits [7–9]. In our study, perioperative mortality and DFS rates of patients with BCLC-B/C HCC were similar to former reports, whereas OS was slightly better.

Although patients with BCLC-B/C HCC often receive surgical treatment, their prognosis is still poor; the main cause of these dismal outcomes is the high incidence of intrahepatic recurrence, which can result from intrahepatic metastasis or multicentric occurrences of new tumors [14–17]. Time to tumor recurrence has been suggested as the key earmark distinguishing between intrahepatic metastases and multicentric occurrences, [17, 18] and the prognosis of patients with multicentric occurrences after curative resection is significantly better than that of patients with intrahepatic metastasis [19]. Histological analysis of HCC recurrence suggests that intrahepatic metastases result in early recurrences, whereas late recurrences appear to arise mainly from multicentric tumors [20]. We therefore believe that early and late post-surgical recurrences in patients with BCLC-B/C HCC might require different management strategies, due to differences in their biological mechanisms. In the present study, we chose 2 year after resection as the cut-off between early and late intrahepatic recurrences, to evaluate their predictive factors, treatment, and prognoses.

In our series, the prognosis of BCLC-B/C HCC patients who did not develop recurrence was significantly better than that of the corresponding recurrence group; and for those who developed recurrences, those with late recurrences had higher 1-, 3-, and 5-year survival rates than did those with early recurrences. Further, 41.3% of the BCLC-B/C HCC patients’ recurrences were late-recurring, which suggests that the postoperative recurrence mechanisms of more than 40% of these patients were multicentric occurrence, which differs from the previous reports [17]; and implies that appropriate treatment strategies for recurrent tumors could vary according to the interval after hepatectomy. Multivariate analysis showed that ≥ 3 tumors, tumors ≥10 cm and vascular invasion were independent risk factors for both recurrence and poor OS in these patients. While these independent risk factors are the characteristics of BCLC-B/C HCC patients and verify the high recurrence rate of these patients. This coincidence among factors conditioning recurrence and survival also confirms the effect of tumor recurrence on OS, and indicates that radical *vs.* palliative treatment should cautiously selected.

Although the OS of BCLC-B/C HCC patients are significantly shortened by tumor recurrence, the curative treatment for recurrent lesions, including surgery or RFA, improved their OS. Most patients who received curative treatments had late-recurring tumors (65%, 15/23), and their OS was significantly better than those who received palliative or conservative treatment-in fact, did not significantly differ from those who were recurrence-free. Moreover, 17.8% (13/73) of patients with late-recurring BCLC -B/C HCC underwent re-resection, a higher percentage than for early-recurring patients (4.8%, 5/104), and even higher than the reported re-resection rate of patients with BCLC 0–A HCC patients with recurrence (7.9–14.7%) [17, 21, 22]. We believe that this phenomenon may partly reflect the multicenter occurrence behind most late recurrences, which is characterized by less malignancy, smaller tumors and more confined location than intrahepatic metastasis. Better outcomes for the late-recurring subgroup might also be the result of longer interval time after hepatectomy allowing better recovery of liver function and increased residual liver volume, which would improve the likelihood of an R0 resection with minimal parenchymal sacrifice and the flattest cut surface. Improved hepatectomy technique and understanding of liver anatomy over time might also lead to more successful surgeries for the late-recurring subgroup. In addition, for those who lose their chance to undergo curative treatment, palliative treatment can still lead to better OS than conservative treatment.

Unlike the late-recurrence subgroup, only a small proportion of patients with early-recurring tumors had the opportunity to receive radical treatment. Notably, the OS of early-recurring patients who underwent curative treatment did not significantly differ from those who underwent palliative treatment only. This is apparently reflected in the different prognoses between the early- and late-recurrence subgroups, and indicates the need for different postoperative surveillance, prevention, and management strategies for these two recurrence patterns. Most early-recurrence tumors arise from intrahepatic metastases that are highly malignant and progress rapidly, which results in patients’ poor prognosis and lack of response to curative treatments. At present, TACE is a widely used method of treating intrahepatic tumor recurrence [23]. Sorafenib, as an oral serine/threonine kinase and tyrosine kinase receptor inhibitor, can also be used for HCC recurrence treatment, with safety and effectiveness [24]. Still, we saw better outcomes for the early-recurrence patients in our series who received palliative treatment (including TACE and sorafenib) than for those who received conservative treatment only. Thus, for BCLC-B/C HCC patients with early recurrence, palliative treatment should be considered as the first-line treatment; curative treatments seem to be inappropriate.

In conclusion, time to recurrence and feasibility of curative treatment are the best determinants for the prognosis of HCC patients and should be carefully considered when choosing optimal therapies and strategies in postoperative surveillance, prevention, and management. Curative treatments may prolong overall survival of patients with late recurrences, but should be avoided for those with early recurrences.

This study is subject to the limitations inherent in retrospective work, such as observation data collected at the specific point; censored data due to loss of visits; difficulty in obtaining some clinicopathological data such as the severity of cirrhosis or microvascular invasion; and unavoidable choice bias in the OS analysis. In addition, the mechanisms of intrahepatic metastasis *vs.* multicentric occurrence in BCLC-B/C HCC recurrence clearly require further study. Our study was also limited by the confined sample size, which made a truly matched study or subgroup analysis unfeasible. A larger, multi-center study of patients from a multi-geographic patient base would be more conclusive.

## MATERIALS AND METHODS

### Patients

From January 1989 to October 2011, 1050 consecutive patients with HCC underwent partial hepatectomies with curative intent at our institution, of whom 397 patients were identified from a prospectively maintained database and included in this trial by the following criteria: (a) pathologically diagnosed HCC, clinically confirmed as BCLC stage B or C; (b) treated with surgical resection as a first-line treatment modality, performed as described below (under “Surgical procedure”); and (c) had no previous or simultaneous multiple primary malignancies. We excluded patients who (a) had received other treatment (such as liver transplantation); or (b) whose recorded clinicopathological parameters (such as preoperative liver function grade or tumor differentiation status) were unclear.

### Definitions

BCLC stage B is defined as intermediate-stage HCC, with Child–Pugh grade A or B liver function, and large, multifocal tumors (cumulative tumor size >5 cm or number of tumor >3 nodules), but with no cancer-related symptoms, macrovascular invasion, or extra-hepatic spread [14].

BCLC stage C is defined as advanced-stage HCC, in which tumors have spread beyond the liver, show vascular invasion and/or patients show mild cancer-related symptoms (Eastern Cooperative Oncology Group [ECOG] grades 1–2) [14].

Curative therapy for HCC was defined as treatment with intent to cure, such as RFA and surgical resection. Surgical resection was defined as complete macroscopic removal of tumors, including tumor thrombi, without exposure of tumor cells on the cut surface, and which conforms to the surgical procedure described below.

Palliative therapy was defined as treatment with intent to cytoreductive tumor tissue or inhibit tumor growth but unlikely to produce a clinical cure, such as Trans-arterial chemoembolization (TACE) and sorafenib.

Conservative treatment was defined as treatment not considered being curative or palliative therapies, with intent to alleviate symptoms such as pain, or improve liver function or even only provide the best supportive care.

Perioperative mortality was defined as death within 30 days after surgery.

Early recurrence is defined as tumor recurrence within 2 year after surgery. Late recurrence was defined as recurrence later than 2 year after surgery. Overall survival (OS) was defined as the interval between surgery and death or the last date of follow-up.

Disease-free survival (DFS) was defined as the date from resection to the date when tumor recurrence was diagnosed; cases where recurrence was not diagnosed at the end of this study were censored on the date of death or the date of last follow-up.

### Surgical procedure

Patients’ preoperational conditions were reassessed to ensure that they conformed to the surgical resection criteria outline in the Updated Standards for the Diagnosis and Treatment of Primary Liver Cancer, [25] which include normal general condition with no obvious pathological changes of heart, lung, kidney and other important organs; normal liver function or Child–Pugh Class A preoperative liver function with or without short-term liver protecting therapy; liver reserve function in normal range; and no unresectable extrahepatic HCC metastasis.

Surgical planning and operations were performed by similar surgical techniques. The resection area was determined with the intent of surrounding the tumor at its deepest portion, while combining minimal parenchymal sacrifice and the flattest cut surface. Parenchymal transection was performed using the Cavitron ultrasonic aspiration system (CUSA) or Ligasure. Hepatectomy was combined with RFA for patients with multiple tumors ≤ 2cm in diameter. Patients with vascular tumor thrombi received thrombectomies. Hilar dissection with intent to perform systemic lymphadenectomy was not routinely performed except in patients suspected to have metastatic lymph nodes on preoperative magnetic resonance imaging (MRI) or computed tomography (CT). The liver resections were recorded according to nomenclature described in the Brisbane 2000 system [26].

### Clinicopathologic features

Clinical data were collected, including age, sex, and such laboratory test results, including alanine aminotransferase (ALT) aspartate aminotransferase (AST), total bilirubin, albumin concentration, prothrombin time (PT), hemoglobin, serum hepatitis B virus (HBV) surface antigen level, hepatitis C virus (HCV) antibody level and serum alpha-fetoprotein (AFP). Histopathologic information regarding tumor number, cumulative tumor size, vascular invasion, extra-hepatic spread and cirrhotic change in the background liver was recorded. Liver function was assessed by the Child–Pugh scoring system. Tumor differentiation was graded by the Edmondson grading system [27].

### Follow-up and treatment for tumor recurrences

All patients were regularly followed up in the outpatient department and prospectively monitored for recurrence by a standard protocol that included measurement of serum AFP, ultrasound examination and contrast CT or MRI examination. Patients were followed up every 3 months during the first postoperative year and at least every 6 months thereafter. Abdominal CT or MRI scans were performed every 6 months. Recurrence was diagnosed based on the typical imaging appearance on CT or MRI. A positron emission tomography (PET) scan was suggested in patients who underwent surgery unless new suspicious lesions were detected by CT or MRI.

Patients diagnosed with HCC recurrence could receive further treatment. If the recurrence was localized and patients had preserved liver function, re-resection or RFA was suggested; if the recurrence was diffuse and patients still had preserved liver function (Child-Pugh Class A or B), TACE was selected; if the patient's general situation or liver function was poor, only conservative treatment was suggested.

### Statistical analysis

The clinical and pathological factors of the groups were compared using Fisher's exact test or Pearson's χ^2^-test, as appropriate. Survival rates were calculated by the Kaplan-Meier method. Cox regression analysis was used to identify independent risk factors with hazard ratio and 95% confidence interval. *P* value <0.05 was considered significant. Data analysis was performed using SPSS 19.0 software (IBM Corp., Armonk, NY, USA).

## Abbreviations

AFP: alpha-fetoprotein
ALT: alanine aminotransferase
AST: aspartate aminotransferase
BCLC-B: Clinic Liver Cancer Stage B
BCLC-C: Clinic Liver Cancer Stage C
CUSA: Cavitron ultrasonic aspiration system
CT: Computed Tomography
DFS: Disease-free survival
HBV: hepatitis B virus
HCV: hepatitis C virus
HCC: Hepatocellular Carcinoma
MRI: Magnetic Resonance Imaging
OS: Overall Survival
PET: positron emission tomography
PT: prothrombin time
RFA: Radiofrequency ablation
TACE: Trans-arterial chemoembolization.
